# A rare infection in the tropics that is not uncommon in cases of chronic granulomatous disease

**DOI:** 10.1099/acmi.0.000039

**Published:** 2019-06-26

**Authors:** Rakesh Bansie, Soeradj Harkisoen, Varsha Lachman, Esther Lai A. Fat, Navin Ramdhani, Jan A. M. van Laar

**Affiliations:** ^1^ Department of Internal Medicine, Academic Hospital Paramaribo, Paramaribo, Suriname; ^2^ Department of Dermatology, Academic Hospital Paramaribo, Paramaribo, Suriname; ^3^ Intensive Care Unit, Academic Hospital Paramaribo, Paramaribo, Suriname; ^4^ Departments of Clinical Immunology and Internal Medicine, Erasmus University Medical Center Rotterdam, Rotterdam, The Netherlands

**Keywords:** chronic granulomatous disease, *Chromobacterium violaceum*, septicaemia

## Abstract

*
Chromobacterium violaceum* is a rare cause of infection in immunocompromised patients in the tropics with a spectrum of disease manifestations, including severe disease. Early identification of this micro-organism is essential for appropriate management. We present a case of *C. violaceum* septicaemia in a patient with chronic granulomatous disease.

## Introduction

Chronic granulomatous disease (CGD) is a rare primary immunodeficiency disease that results from phagocyte dysfunction due to a defective NADPH oxidase system and results in an impaired respiratory burst [1]. It is an X-linked inheritance in 65–70 % of cases and is autosomal recessive in the remaining cases [1]. CGD patients are susceptible to infections with catalase-positive micro-organisms and fungi of e.g. the lungs, lymph nodes, liver and skin [1, 2]. Commonly reported micro-organisms include *Aspergillus* species, *Staphylococcus aureus *, *Burkholderia cepacia *, *Serratia marcescens* and *Nocardia* species [2]. The dysregulated inflammatory response also results in granuloma formation and other inflammatory disorders, such as colitis and lupus-like skin lesions [1, 3]. CGD is diagnosed by means of defective respiratory burst responses detected with dihydrorhodamine or nitroblue tetrazolium tests [2]. Antibiotic prophylaxis consisting of trimethoprim/sulfamethoxazole and intraconazole is advised [4].

*Chromobacterium violaceum* is a rare Gram-negative facultative anaerobic bacillus that is mainly found in soil and water in tropical and subtropical climates [5]. It is catalase- and oxidase-positive and is usually susceptible to carbapenems, quinolones, trimethoprim/sulfamethoxazole and aminoglycosides [5, 6]. This micro-organism can be highly pathogenic in immunocompromised patients predominantly suffering from neutrophil dysfunction, such as in cases of CGD [7]. Most clinical data are based on observations from fewer than 200 cases [5, 8]. Of all patients with *C. violaceum* infections, 8.5 % had CGD [5]. Clinical symptoms include pulmonary abscesses and fatal septicaemia. Infections result from breaks in the skin together with exposure to soil and water. The mortality rates for *C. violaceum* infections vary between 53–80 %, with a higher mortality in disseminated disease [7]. We present a case of *C. violaceum* septicaemia in a patient with CGD who was on vacation in Suriname. Written consent was obtained from the patient and approval was obtained from the Ethical Committee of our hospital. The rarity of *C. violaceum* infections and the challenges associated with management of these infections in patients with CGD warrants reporting of such cases.

## Case report

**Fig. 1.**
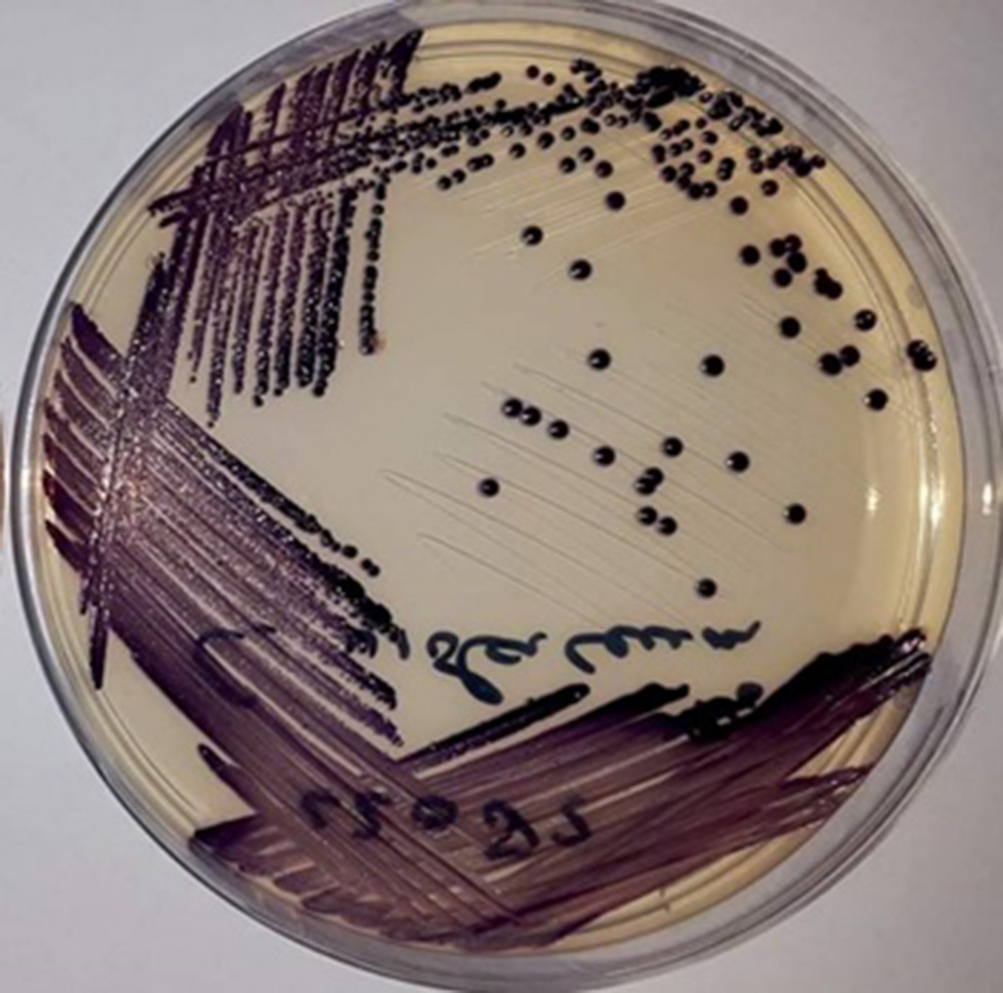
Blood culture on Petri plate of *C. violaceum *. Image courtesy of Bryan Mangroe, Department of Microbiology, Academic Hospital Paramaribo, Suriname.

In 2016 a 37-year-old man with X-linked CGD and lupus-like skin lesions presented with red discoloration of the skin around the xiphoid process, fever and heavy pain in the abdomen that had begun 3 days earlier. He was on vacation in Suriname and had visited a creek recreational area a few days earlier. His initial diagnosis was necrotizing fasciitis. He appeared to be suffering from sepsis, with a blood pressure of 130/90 mmHg, a heartbeat of 120 b.p.m., a respiratory rate of 20 breaths min^−1^, a peripheral oxygen saturation of 96 % and a temperature of 39 °C. His abdomen was tender without resistance. The only plasma abnormalities were creatinine 117 μmol l^−1^ (60–110 μmol l^−1^), sodium 130 mmol l^−1^ (132–148 mmol l^−1^), C-reactive protein 22.1 mg dl^−1^ (0.0–0.5 mg dl^−1^), leukocytes 20.0×10^9 ^l^−1^ (4.5–11.0×10^9 ^l^−1^) and thrombocytes 147×10^9 ^l^−1^ (150–400×10^9^ l^−1^). He started with amikacin, clindamycin and flucloxacillin. A necrotomy was performed the next day. A computed tomography (CT) scan of the thorax and abdomen revealed pre-broncheal nodular opacities and hepatosplenomegaly and no characteristic findings for necrotizing fasciitis. Within 48 h the patient developed respiratory failure and haemodynamic instability, for which he was transferred to the intensive care unit (ICU) for mechanical ventilation and intravenous vasopressors. He had progressive renal failure and elevated liver enzymes and developed leukocytopenia and trombocytopenia; creatinine 372 μmol l^−1^, aspartate aminotransferase 100 U l^−1^, alanine aminotransferase 102 U l^−1^, lactate dehydrogenase 352 U l^−1^, leukocytes 3.8×10^9^ l^−1^, thrombocytes 79×10^9^/L. A repeat CT scan of the thorax and abdomen showed progressive ground glass opacities from the lower lobe of the lungs, pleural effusion, ascites and hepatosplenomegaly with liver abscesses. The wound, blood and faecal cultures turned positive for *C. violaceum *, with sensitivity to gentamicin, doxycyclin, ciprofloxacin and trimethoprim/sulfamethoxazole (Fig. 1). The patient’s antibiotics were switched to ciprofloxacin and meropenem because of progressive renal impairment. Voriconazole was empirically added to the regime. Ancillary testing for *Aspergillus* could not be performed. No fungi were later cultured from the different samples. Prednisone was added due to secondary adrenal insufficiency in septic state. Five days after presentation the patient started with continuous veno-venous haemofiltration (CVVH) due to progressive renal failure with oliguria and generalized edema. This was discontinued 5 days later after renal function improvement. The mechanical ventilation could also be discontinued afterwards. He was dismissed from the ICU after a total of 14 days. Further care and rehabilitation continued in strict isolation due to a multi-resistant *Acinetobacter baumanni* in inventory sputum cultures. After a total of 21 days the patient could be discharged from the hospital and he returned to the Netherlands.

## Discussion

Here we describe the clinical course of a CGD patient visiting Suriname who acquired a severe *C. violaceum* infection. Infection with *C. violaceum* is rare in humans and can have a fulminant presentation with systemic metastases and a high mortality rate. CGD contributes to nearly 8.5 % of all reported *C. violaceum* cases [5]. Patients with CGD (especially children) are susceptible to *C. violaceum *. The potential severity of *C. violaceum* infections is reflected by the described severe clinical course. The pulmonary lesions initially progressed and were considered to be of infectious origin. A poor outcome is seen in patients treated with an inadequate antibiotic regime or with disseminated infection or bacteraemia [9]. The optimal antibiotic regime and the duration are yet to be determined, but apparently the antibiotic regime should include one of the following antibiotics: chloramphenicol, gentamicin, fluoroquinolones, tetracyclines, carbapenems, trimethoprim/sulfamethoxazole, or semisynthetic penicillins [10]. Our patient also received voriconazole. Fungal infections can be indistinguishable from bacterial infections, especially in limited-resource settings such as Suriname. Antifungal treatment should readily be considered in CGD patients whose treatment with antibiotics is failing. With CGD, a multidisciplinary approach is warranted in such cases and expert consultation should readily be considered and should be readily available. We advise patients with CGD to avoid exposure to brackish waters and walking barefoot outdoors, and to adopt caution with skin wounds when outdoors. *C. violaceum* infection is an important differential diagnosis in CGD patients who present with skin lesions and fever, and have a history of travelling to the tropics.
